# Supplementary material for: A Retrospective Cohort Study of a Newly Proposed Criteria for Sporadic Creutzfeldt–Jakob Disease
Source: Diagnostics (Basel). 2024 Oct 30;14(21):2424. doi: 10.3390/diagnostics14212424 (PMC11545003; doi:10.3390/diagnostics14212424)
Supplement: Supplementary file 1 [file diagnostics-14-02424-s001.zip › Supplementary Tables S1 and S2.pdf]

Table S1: WHO criteria and Hermann's criteria

WHO criteria for sporadic CJD (1998)

Definite:

Diagnosed by standard neuropathological techniques; and/or

Immunocytochemically and/or Western blot confirmed protease-resistant prion protein and/or

Presence of scrapie-associated fibrils.

Probable:

I + two of II and typical EEG or

I + two of II and positive CSF 14-3-3 protein and a clinical duration to death < two years

Routine investigations should not suggest an alternative diagnosis.

Possible:

I + two of II and no EEG or atypical EEG + duration < two years

==

I Rapidly progressive cognitive impairment

II A. Myoclonus

B. Visual or cerebellar disturbance

C. Pyramidal or extrapyramidal signs

D. Akinetic mutism

==

Zeidler, M.; Gibbs, C.J.; Meslin, F. WHO Manual for Strengthening Diagnosis and Surveillance of Creutzfeldt–Jakob Disease [Online]; World Health Organization, 1998; p. 47. Available online: https://iris.who.int/handle/10665/66394.

Hermann criteria for sporadic CJD (2021)

Definite:

Progressive neuropsychiatric syndrome AND neuropathological or immunocytochemical, or biochemical confirmation

Probable:

I + two of II and typical EEG or

I + two of Il and typical brain MRI or

I + two of Il and positive CSF 14-3-3 protein or

progressive neuropsychiatric syndrome and positive RT-QuIC assay in CSF or other tissues

+ exclusion of other causes in complete diagnostic workup

Possible:

I + two of II + duration < two years

==

I Rapidly progressive cognitive impairment

II A. Myoclonus

B. Visual or cerebellar disturbance

C. Pyramidal or extrapyramidal signs

D. Akinetic mutism

==

Hermann, P.; Appleby, B.; Brandel, J.P.; Caughey, B.; Collins, S.; Geschwind, M.D.; Green, A.; Haïk, S.; Kovacs, G.G.; Ladogana, A.; et al. Biomarkers and Diagnostic Guidelines for Sporadic Creutzfeldt–Jakob Disease. *Lancet Neurol.* **2021**, *20*, 235–246. DOI:10.1016/S1474-4422(20)30477-4.

Table S2: Categorisation of non-prion disease cases

The 146 non-prion disease cases included the following diseases: primary or secondary epilepsy (including post convulsive status encephalopathy and non-convulsive status epilepticus), autoimmune encephalitis (including Hashimoto's encephalopathy, anti-NMDA receptor encephalitis, and immune-mediated cerebellitis), limbic encephalitis, encephalopathy, autoimmune-mediated encephalopathy, neurodegenerative disease (Alzheimer's disease, spinocerebellar degeneration, frontotemporal dementia, Lewy body dementia, corticobasal degeneration, progressive supranuclear palsy, multiple system atrophy, amyotrophic lateral sclerosis, Parkinson's disease, Parkinson's syndrome, and motor neuron disease with dementia), paraneoplastic syndrome (limbic encephalitis due to paraneoplastic syndrome and encephalitis due to paraneoplastic syndrome), metabolic encephalopathy (alcohol related disorders; Wernicke encephalopathy; folic acid deficiency; hepatic encephalopathy; hypoxic encephalopathy; hypoglycemic encephalopathy; and mitochondrial myopathy, encephalopathy, lactic acidosis, and stroke-like episodes), drug-induced parkinsonism, insomnia, progressive multifocal leukoencephalopathy, primary cerebral malignant lymphoma, post-transplant lymphoproliferative disease, intravascular large B-cell lymphoma, multiple brain metastases with lung cancer, sequelae after encephalitis, idiopathic hypertrophic meningitis, chorea with polycythemia vera, shunt dysfunction after shunt surgery for normal pressure hydrocephalus, spastic paraplegia, neurosyphilis, mental illness (atypical psychosis, obsessive-compulsive disorder, depression, organic psychosis, severe depression with psychotic symptoms, sub-confusion, catatonia due to depression, mental retardation, and schizophrenia), vascular dementia, and acute cerebral infarction. As mentioned above, various diseases and pathological conditions have been observed in non-prion diseases.
